# A cerebellopontine angle metastatis of a male breast cancer: Case report

**DOI:** 10.1016/j.amsu.2022.103421

**Published:** 2022-03-01

**Authors:** Yassine Tahrir, Abderazak Bertal, Sara Mawhoub, Marouane Makhchoune, Khadija Ibahiouin, Abdelhakim Lakhdar

**Affiliations:** Neurosurgery department, University Hospital Center IBN ROCHD, Casablanca, Morocco

**Keywords:** Male breast cancer- cerebellopontine angle- metastasis -case report

## Abstract

Male breast cancer is rare, less than 1% of men's cancers. The tumors occurring in the cerebellopontine angle remain a rare entity. Features suggestive of metastasis are acute onset, rapid progression of symptoms. We report a case of a 72-year-old man had a mastectomy and an axillary lymph node dissection for a breast cancer 22 years prior to this report. The patient was admitted with deterioration of level of consciousness with intracranial hypertension syndrome. The magnetic resonance imaging showed a cystic lesion in the left cerebellar hemisphere and the prepontine cistern. We proceeded to a large tumor resection. On the follow up, the patient presented a delayed emergence. A CT scan showed a small hematoma at the surgical site and triventricular hydrocephalus for which the patient underwent a ventriculoperitoneal shunt. This is the first described cerebellopontine angle metastasis of a male breast cancer and the first described case of a metastatic triple hormone negative breast cancer to the brain.

## Highlights

• A 72 years old man had a mastectomy and an axillary lymph node dissection for a breast cancer 22 years prior to this report. He was brought to our emergency for intracranial hypertension syndrome with disorders of consciousness.
• The magnetic resonance imaging showed a cystic lesion in the left cerebellar hemisphere compressing the pons, the acoustic-facial nerve bundle, with a peripheral contrast enhancement.
• This is the first described cerebellopontine angle metastasis of a male breast cancer and the first described case of a metastatic triple hormone negative breast cancer to the brain.

## 1 Introduction

Male breast cancer represents less than 1% of total breast cancers and less than 1% of men's cancers [1]. The major risk factors for the development of male breast cancer include advancing age, hormonal imbalance, radiation exposure, and a family history of breast cancer as well as some gene mutations. Brain metastases occur in up to 24% of breast cancer [1,2]. In male patients, it's even rarer. Only 7 case reports of male patients with brain metastases of a breast cancer were reported. Most of them were multiple (supra and infratentorial) or unique supratentorial metastasis [2,3]. This report presents the case of a cerebellopontine angle unique metastasis of a breast cancer in a male patient treated 22 years ago.

## 2 Case report

**Fig. 1:**
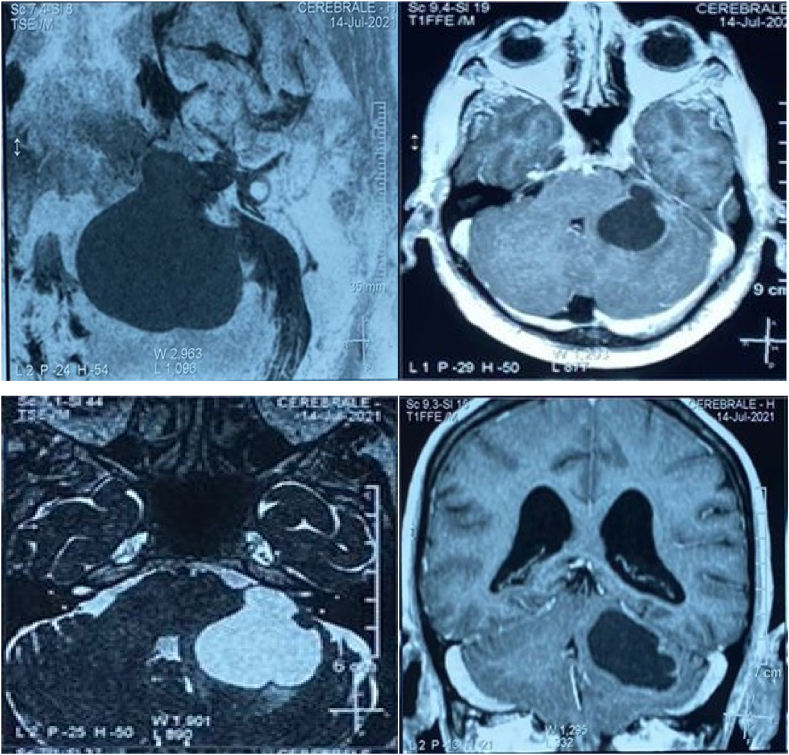
Axial and coronal MRI imaging scan showing a cystic lesion depending on the left cerebellar hemisphere, hyposignal in T1 and T2 hypersignal, enhanced peripherally after contrast injection, measuring 32 × 35 mm occupying the prepontic cistern and compressing the cerebral peduncles and the 4th ventricle.

A 72 years old man had a mastectomy and an axillary lymph node dissection for a breast cancer 22 years prior to this report. The tumor was a moderately differentiated adenocarcinoma with triple hormone negative (ER, PR, HER2). The patient was also treated with adjuvant chemotherapy, radiation therapy. Nine years ago, the patient presented with a tumor recurrence, treated with chemotherapy and radiation therapy. The patient also had a history of minor head trauma resulting in a bilateral subdural hematoma three years ago, treated surgically. Two years ago, the patient presented with progressive hearing loss. A year and half later, he started to complain of headache, vomiting, gait disorder and dysphagia. At admission, his GCS score was 14/15 (E4V4M5). Neurological examination revealed a right hemiparesis and a peripheral facial nerve palsy. The magnetic resonance imaging showed a cystic lesion in the left cerebellar hemisphere and the prepontine cistern (3.2× 3.5 cm) compressing the pons, the acoustic-facial nerve bundle, the fourth ventricle and the cerebellar peduncles, with a peripheral contrast enhancement (Fig. 1). To relieve the symptoms and confirm the pathology, we proceeded to a large tumor resection.

**Fig. 2:**
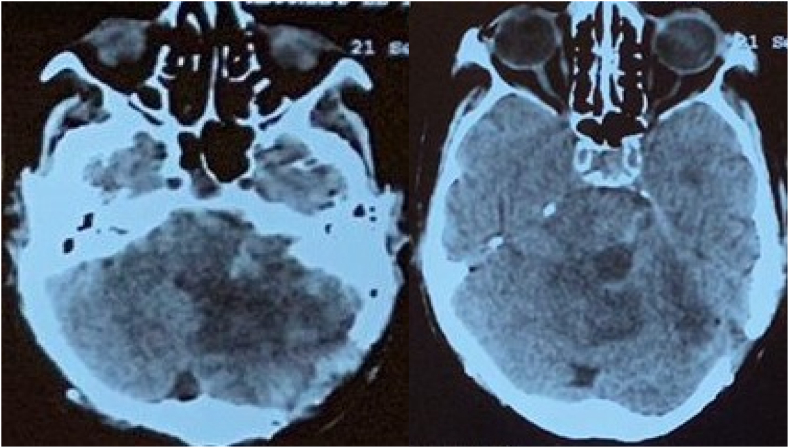
Immediate postoperative CT scan showing oedemato-hemorrhagic remodeling at the surgical site.

The intervention was performed by our chief resident under general anesthesia a wide occipital craniotomy was performed and we proceeded to a large tumor resection with dural plasty. On the follow up, the patient presented a delayed emergence. A CT scan showed a small hematoma at the surgical site and triventricular hydrocephalus (Fig. 2) for which the patient underwent a ventriculoperitoneal shunt. The patient regained consciousness and presented no further neurological deficits. He was discharged after 10 days. Through pathological examination, the patient was diagnosed with brain metastasis of a poorly differentiated invasive adenocarcinoma with negative hormone receptors (Estrogen receptor (ER), progesterone receptor (PR).

This case has been reported in line with the 2020 SCARE guidelines [4].

## 3 Discussion

The pontocerebellar angle is a prismatic space containing several noble elements such as the facial nerve, the vestibulocochlerar nerve and the mixed nerves. It is limited in the back by the brain stem and the cerebellar hemisphere. The compression of these elements is at the origin of otological, neurological and vestibular symptoms as well as an intracranial hypertension syndrome due to the compression of the 4th ventricle. The tumors of the pontocerebellar angle (CPA) are dominated by the acoustic neuroma while the metastases represent less than 1%. Features suggestive of metastasis are acute onset, rapid progression of symptoms and seventh and/or eighth nerve deficits. Female breast cancer is a common cause of brain metastases (10–16%) but the tumors occurring in the cerebellopontine angle remain a rare entity [1].

Male breast cancer accounts for 0.6–1% of all breast cancers and about 0.3% of all cancers in men [1]. Men are diagnosed at a median age of 63.3 years [2]. The main risk factors are an advanced age, a family history of breast cancer, the presence of a BRCA mutation (BRCA1 and BRCA2), hormonal factors such as an increased serum estradiol, Klinefelter's syndrome and testicular abnormalities [3]. The risk also significantly increases with increasing body mass index (BMI) [5]. The lifetime risk of developing a breast cancer for a man is approximately 1:833 [6].

Compared with female patients, male patients have higher mortality across all stages [2]. This survival differences may be attributed to older age, later stage at diagnosis, and shorter life expectancy in men [3]. The American Cancer Society estimates about 2650 new cases of invasive breast cancer in men in the United States for 2021 and about 530 deaths [6]. Male breast cancer metastases mainly occur in the bones (62%), followed by the lungs, the liver and skin [7,8].

The incidence of brain metastasis from female breast cancer is around 24% and usually occur in locally advanced tumors [9]. The median time intervals between the diagnosis of breast cancer to identification of brain metastasis is 34 months [9]. Cerebellum and frontal lobes are the most common sites of metastasis [9]. While in male breast cancer, information about brain metastases is lacking. A database search found only 7 cases of male breast cancer with brain metastasis [[10], [11], [12], [13], [14], [15], [16]]. The time intervals between the diagnosis of breast cancer to identification of brain metastasis vary between 1 and 24 years with a median of 7 years [[14], [15], [16]]. In our case, the delay between the breast cancer and the appearance of neurological symptoms was 22 years.

Also, all of the male breast cancers with brain metastases had positive hormonal receptors. Among the described brain metastases, three were with multiple locations, and the rest were unique supratentorial tumors [[10], [11], [12], [13], [14], [15], [16]].

Cerebral MRI in thin-section in T1, T1 after gadolinium injection as well as T2 and gradient echo can reveal small lesions of the CPA [17]. The MRI usually us images much more extended than the clinical symptoms indicate in the case of cerebral metastases [18]. It will also demonstrate large areas of edema around metastatic tumors that may not be found in benign tumors [17].

If the primary site is not identified, we will attempt to obtain histological evidence from the intracranial lesions to identify the primary tumor. If the primary tumor site is found, treatment will be directed at both the intracranial and primary site lesions. In our case, we operated the patient, and we put him in contact with the oncologists for a possible complementary radiotherapy.

The particularity of our case, this is the first described cerebellopontine angle metastasis of a male breast cancer and the first described case of a metastatic triple hormone negative breast cancer to the brain.

## 4 Conclusion

The tumors occurring in the cerebellopontine angle remain a rare entity. Compared with female patients, male patients have higher mortality. The characteristics of a metastasis are the rapid onset of symptoms and nerve deficits. A multidisciplinary consultation meeting between a neurosurgeon and an oncologist is essential in order to make the best therapeutic decision.

## Provenance and peer review

Provenance and peer review Not commissioned, externally peer-reviewed.

## Sources of funding

None.

## Ethical approval

Written informed consent for publication of their clinical details and/or clinical images was obtained from the patient. Ethical approval has been exempted by our institution

## Research Registration Unique Identifying Number (UIN)

None.

## Author contribution

Yassine TAHRIR: writing the paper and Corresponding author. Abderrazak BERTAL: Correcting the paper. Sara MAWHOUB: Correcting the paper. Marouane MAKHCHOUNE: Correcting the paper. Khadija IBAHIOUIN : Correcting the paper. Abdelhakim LAKHDAR: Correcting the paper

## Guarantor

TAHRIR YASSINE

## Declaration of competing interest

The authors declare having no conflicts of interest for this article.
